# Clinical improvement of teens participating in a dbt skills training

**DOI:** 10.1192/j.eurpsy.2023.1517

**Published:** 2023-07-19

**Authors:** I. Ezquiaga Bravo, A. M. Rodríguez, A. Vilar, A. Salvador, M. Biempica, K. M. García, L. M. Martín, S. Batlle

**Affiliations:** INAD, Parc de Salut Mar, BARCELONA, Spain

## Abstract

**Introduction:**

Dialectical behavioural therapy (DBT) developed by Linehan has been shown to be widely effective in improving emotional regulation capacity in patients diagnosed with borderline personality disorder in adults and adolescents, but also for other profiles of emotional dysregulation, even in the general non-clinical population through emotional regulation skills training programs in schools.

**Objectives:**

The objective was to describe psychopathological characteristics and to evaluate clinical outcome variables (self-harm, suicide attempts, admissions and emotional regulation difficulties) in young patients who participated in the DBT skills training group carried out by the child and adolescent psychiatry team of Hospital del Mar (Barcelona) between February 2020 and April 2022.

**Methods:**

Prospective longitudinal study with two evaluations (before starting the group and after finishing it). The clinical variables were evaluated by reviewing the medical records, and the improvement in emotional regulation difficulties was evaluated through the Difficulties in Emotion Regulation Scale (DERS) adaptation to adolescents before and after the intervention.

**Results:**

A total of 36 participants have been referred and assessed to participate in the previously mentioned emotional regulation program. The mean age was 15.6 years (14-17 years old). 100% of the participants were female. All of them met criteria for BPD according to the SCID-II questionnaire; but only 23 patients (63.9%) had BPD as their main diagnosis. 63.9% (n=23) presented psychiatric comorbidities, being 27.8% (n= 10) ADHD, 30.6% (n= 11) substance use disorder and 47.2% (n= 17) eating disorders,

77.8% (n=28) had presented self-injurious behaviour, 52% (n=18) had committed a suicide attempt, requiring hospital admission in 36.2% (n=13) at some point in their lives before the therapy group. In the three months after the end of the group, admissions were reduced to 17% (n=6), suicide attempts to 14.8% (n=5) and non-suicidal self-injurious behaviours to 27.8% (n=10).

The mean score of all participants on the DERS scale was 129.91 points before participating in the DBT skills group and 105 points right after the group finished. Higher scores translate into greater emotional regulation difficulties.

**Conclusions:**

The reduction in DERS scores, self-injurious behaviours, suicide attempts, and admissions was notable for all participants. It remains to add the results of the groups currently in operation and perform the statistical analysis of all the results. It is necessary to continue studying and testing the benefits of DBT both in the clinical adolescent population and in the general child and adolescent population in order to generalize the promising results observed in our sample. At Hospital del Mar, we will continue to expand the DBT program so that more children and adolescents with emotional dysregulation can benefit.

**Disclosure of Interest:**

None Declared

